# Emerging Roles of Chemokines in Cancer Immunotherapy

**DOI:** 10.3390/cancers14153593

**Published:** 2022-07-23

**Authors:** Bernhard Moser

**Affiliations:** Division of Infection & Immunity, Henry Wellcome Building, Cardiff University School of Medicine, Heath, Cardiff CF14 4XN, UK; moserb@cardiff.ac.uk

This series of 15 articles (4 original articles and 11 reviews) is presented by international leaders in chemokine research. All publications focus on the roles of chemokines and their receptors in the context of cancer, either with respect to their pro- or anti-tumor functions. The collection of articles is very timely for two reasons: (1) Current cancer therapy is progressing with increasing speed thanks to the development of immune-checkpoint-targeted therapies. The success of blocking antibodies specific for the inhibitory co-receptors CTLA-4 and PD-1/PD-L1 has unequivocally demonstrated the critical importance of the immune system in cancer control. Consequently, their use represents a game change in current cancer therapy, and (2) chemokine receptors are expressed on both tumor cells as well as immune cells (the primary target of chemokines) present in the tumor microenvironment. Similarly, the tumor milieu is characterized by a mixture of chemokines that varies with the type and stage of cancer. The chemokine system is highly complex, and their net contributions to tumor development and metastasis are context-dependent and may include both tumor-promoting and tumor-inhibiting functions.

The compartment of immune cells in the tumor microenvironment resembles chronically inflamed tissues and healing wounds, and includes both resident and recruited immune cells. Unsurprisingly, chemokines are recognized as key regulators of cancers, as attested by an avalanche of recent studies. Chemokines, reflecting their nom de guerre, control the recruitment and/or tissue retention of immune cells as well as the mobilization of tumor cells that have undergone epithelial–mesenchymal transition. Some chemokines promote tumor growth. In fact, the tumor growth activities of GROα, β and γ were documented before they were shown to be potent chemokines for human neutrophils (now known as CXCL1-3). Finally, chemokines also modulate stromal cell functions, including cytokine and growth factor secretion, often in concert with co-stimulatory factors present in the tumor micro-environment.

The four original research articles represent examples of current tumor-related chemokine research. Chemokines are bi-functional in their ability to interact with their cognate receptors on target cells as well as proteoglycans, such as heparin sulfate (HS), present on cell surfaces and extracellular matrix. One article explored the possibility of using mutated CCL21, a CCR7-specific chemokine engineered to lack the HS-binding portion, to interfere with metastasis of tumor cells to the lymph nodes [1]. Similarly, a second article examined the possibility of using the HS-binding portion of CXCL9 to inhibit the angiogenic properties of diverse growth factors (VEGF, FGF, EGF) [2]. CX3CL1 (fractalkine) is a strong chemoattractant for NK cells. Interestingly, in the microenvironment of oesophagogastric adenocarcinomas, the anti-tumor function of NK cells is inhibited by CX3CL1, suggesting that the therapeutic inhibition of CX3CL1 receptors (CX3CR1) might be beneficial [3]. The fourth paper reveals an unexpected role of PD-L1, one of the two ligands for the inhibitory receptor PD-1 expressed on “dormant”, tumor-specific effector T cells, in modulating anti-tumor immunity. In fact, PD-L1 signaling, together with other factors including chemokines, promotes tumor growth and metastasis in a mouse tumor model [4].

The 11 review articles written by chemokine specialists provide a cross-section of current tumor-related chemokine research and underscore the multiple and in part overlapping functions assigned to chemokines in cancer. Chemokines are instrumental in defining the immune infiltrate, including anti-tumor effector T cells as well as tumor-promoting Treg cells, macrophages, myeloid-derived suppressor cells (MDSC) and neutrophils [5]. On the same line of thought, it has been suggested that certain chemokines could be exploited to turn a “cold” tumor into a “hot” tumor characterized by T-cell infiltrates that could be targeted by immunomodulatory agents [6]. Several reviews focus on specific types of cancer. For instance, one discussion centers on cutaneous melanoma and the chemokines associated with this type of disease [7], whereas another review looks at glioma and the chemokine receptors that may predict responsiveness to treatment and disease outcome [8]. Mogamulizumab is an approved antibody for the treatment of patients with adult and cutaneous T-cell lymphoma. This antibody selectively binds to CCR4 on T-cell lymphomas and induces their elimination via antibody-mediated cellular cytotoxicity (ADCC) [9]. CXCR4 is one of the best-studied chemokine receptors associated with tumor growth and metastasis, as demonstrated in a separate review dedicated to CXCR4 in breast cancer [1]. This chemokine receptor is also the topic of a discussion focusing on targeting CXCR4 to improve tumor immunotherapy [10]. Another important topic of current cancer research is inhibitory cells present in the tumor microenvironment that may be targeted to alleviate their negative effects on anti-tumor immune responses. For instance, and following the lead of mogamulizumab (see above), one article discusses the merits of ADCC-active antibodies specific for CCR8, a chemokine receptor selectively expressed on intratumor Treg cells [11]. Similarly, MDSCs express CXCR2, and methods of targeting this chemokine receptor to neutralize the immune-inhibitory effects of MDSCs in the tumor microenvironment are explored [12]. Furthermore, neutrophils in the tumor microenvironment correlate with tumor progression and an adverse disease outcome. One review summarizes current research investigating the role of chemokines in the recruitment and function of intratumoral neutrophils [13]. The CXCL13/CXCR5 axis is instrumental in controlling humoral responses in secondary (and tumor-associated) lymphoid tissues. The option of targeting this pathway to augment the effect of immune checkpoint inhibitors is discussed [14]. Finally, a timely article reminds us of the multiple and still ill-defined functions of virus-derived chemokines and chemokine receptors in oncogenic pathways and tumor development [15].

The impressive range of investigations led by leaders in chemokine research is underscored by the long list of ongoing (or planned) clinical trials. Novel chemokine-based reagents are tested either alone as mono-therapies or more frequently in combination with approved therapies in the settings of all major types of cancers. Indeed, there is reason for optimism. Still, chemokines and their receptors fulfill essential functions in all aspects of immune processes, including those related to physiology (hematopoiesis, immune defense, tissue health) and pathophysiology (chronic inflammation, allergy, cancer), suggesting that immune-related adverse events may limit the utility of some of these novel, chemokine-based reagents in cancer therapy.
